# Performance of teenaged action video game players on the Developmental Eye Movement and King-Devick tests

**DOI:** 10.1371/journal.pone.0324862

**Published:** 2025-06-18

**Authors:** Marc Argilés, Cristina Rovira-Gay, Luis Pérez-Mañà, Bernat Sunyer-Grau, Liat Gantz

**Affiliations:** 1 Centre for Sensors, Instruments, and Systems Development (CD6). Universitat Politècnica de Catalunya - BarcelonaTech (UPC). Campus Terrassa. Rambla de Sant Nebridi, Terrassa, Barcelona, Spain; 2 Universitat Politècnica de Catalunya - BarcelonaTech (UPC). Campus Terrassa. Rambla de Sant Nebridi, Terrassa, Barcelona, Spain; 3 Department of Optometry and Vision Science, Jerusalem Multidisciplinary College, Jerusalem, Israel; The University of Tennessee at Chattanooga, UNITED STATES OF AMERICA

## Abstract

Action video game (AVG) players have been reported to demonstrate improved perceptual, cognitive, and motor skills, as well as enhanced eye movements such as saccade latency and accuracy. This study examined if these improvements are also observed in two common clinical eye movement assessments, the Developmental Eye Movement (DEM) and King–Devick tests. Ninth and tenth graders (15–16 years old) without learning disabilities or oculomotor dysfunctions, and who do not regularly play ball sports were tested in the DEM and King–Devick tests in random order. Those playing AVG ≥ 5 hours per week were included in the AVG group, and those playing ≤ 1 h per week were included in the non-video game group (NVG). Participants were asked to read quickly while reading speed and errors were recorded. Two DEM subtests were read vertically and one subtest was read from left to right. All King–Devick subtests were read from left to right. Forty participants (20 AVG, 20 NVG, 15 girls, mean age: 15.7 ± 0.1 years) were included. AVG players were significantly faster in their horizontal DEM times (AVG: 26 ± 5, NVG: 34 ± 7 sec, p < 0.001) and King–Devick times (AVG: 14 ± 2, NVG: 16 ± 4 sec, p = 0.034), but not in their vertical DEM times (AVG: 26 ± 3, NVG: 28 ± 5 sec p = 0.063). King-Devick and horizontal DEM times were significantly correlated for the entire sample (p < 0.01). Teenagers who regularly played AVGs performed better on the DEM and King–Devick tests, indicating that regularly playing this genre of AVGs may enhance oculomotor skills and visual processing speed. Clinicians should take this into account when interpreting results of these clinical tests.

## Introduction

Video games belong to a complex and constantly evolving segment of modern society. Playing video games is a daily activity for a large number of individuals, with children and adolescents being the largest fragment of this group [1,2]. Society’s global concern for video games highlights the dangers of addiction, increased violence, depression, social problems, and poor academic performance [3,4]. Although we cannot underestimate the addiction problems caused by video games, regularly playing AVG still has some advantages regarding cognitive, motivation, emotional, and social aspects [5,6]. Several studies describe the cognitive, [7] perceptual, [8] and motor [9] benefits of action video games (AVG), which have been utilized to improve visual acuity in amblyopia, [10,11] brain injuries, [12] and reading performance in dyslexia [13–15]. Among the genres of videogames (i.e., Sports, Action, Multi-Online Battle Arena, Role-Playing Games, Simulators, Musical, Adventures), AVG requires rapid response times and includes situations with high perceptual, cognitive, and motor loads [16]. AVGs require constant selection among multiple plans, and coordination between focused attention for accurate aim as well as divided attention to detect newly appearing game enemies [6]. Thus, AVG players have been shown to have improved (shorter) saccadic reaction times for both voluntary and reflexive saccadic movements and higher maximum speeds of saccades compared with non-videogame players (NVG) [17]. AVG players have also been shown to be faster in executing saccadic eye movements compared to NVG players [18]. Further, high-level AVG experience is associated with different fixational eye movement patterns during videogames [19,20].

Research has shown that enhanced eye movement performance is linked to better outcomes in activities requiring rapid visual processing, such as reading efficiency, sports performance, and even driving safety [21–24]. Faster and more accurate saccadic movements contribute to improved visual search efficiency, reducing reaction times in tasks that demand quick decision-making, such as emergency response situations and aviation [25]. In addition, the cognitive benefits associated with enhanced eye movement control, such as improved working memory and attentional control, may extend to academic and professional environments that require high levels of concentration and multitasking [26,27].

However, eye-tracking technology is not commonly used in clinics. The typical tests to evaluate eye movement performance are the Developmental Eye Movement (DEM), [28] and the King–Devick tests, [29] which are related to eye movements and visual processing [30,31]. The DEM test is a clinical assessment that evaluates the efficiency of eye movements, particularly eye tracking and fixation [32]. The DEM is especially relevant for individuals with reading difficulties or other visual processing challenges [33]. The King–Devick test, which is similar to the DEM test, identifies impairments in saccadic eye movements and other aspects of visual processing that are often assessed with clinical conditions such as brain concussion, [31] or mild cognitive impairments such as Alzheimer’s disease [34]. Both tests assess eye movements when observers are requested to quickly read a sequence of numbers.

Although previous studies have documented improved oculomotor performance, such as saccade accuracy or latency in AVG players, it is unknown if these improvements are translated to performance on clinical tests such as the DEM and King–Devick. Research have shown that a better performance in eye movement performance in the DEM and King-Devick test are related. This study evaluated the performance of teen-aged AVG players on the DEM and King–Devick tests, which could have important implications for clinical practice.

## Materials and methods

### Participants

This study followed the tenets of the Declaration of Helsinki and was approved by the Ethics Committee of Mútua de Terrassa (Terrassa, Spain), (ref P/22–042/). Measures were conducted between 01/02/2024–30/04/2024. All legal guardians were informed about the nature of the study and signed a written informed consent form before commencement of the study. Inclusion criteria included participants between age 15 and 16 years, corrected visual acuity of at least 0.05 LogMAR (0.90 decimal) or better in the two eyes at distance and near, no history of ocular pathology or ocular surgery, absence of strabismus determined with the prism- neutralized cover-uncover test at distance and near, near point of convergence less than 7 cm of break point and 5 cm of recovery point, and normative monocular amplitude of accommodation based on the participants’ age (Hofstetter formula), [35] measured using the push-up method. Participants with any diagnosis of learning problems such as dyslexia, Attention-Deficit/Hyperactivity disorder (ADHD), or autism, as reported by the legal guardian were excluded. In addition, individuals who reported regularly playing sports for three or more hours per week, particularly ball sports such as soccer, basketball, tennis, ping-pong, or badminton, were excluded, as these activities enhance oculomotor response [36–38].

### Group classification

Classification to the AVG and NVG video game groups was determined based on the participants’ self-reported history of video game play assessed using a questionnaire. The questionnaire included a list of commercial videogames and a question related to weekly play duration in the prior six months (less than 1h, between 3 and 5h, and more than 5 hours) [39]. AVG players were defined as participants who reported playing only action video games for ≥5 hours per week and ≤ 1 h in other video game genres. NVG were defined as participants who reported playing ≤ 1 h per week in action video games or other video game genres. Only participants who met the questionnaire inclusion criteria (AVG or NVG) were included in this study. Commercial videogames included as AVG were Call of Duty, Halo, Apex Legends, Half-Life, Overwatch, Counterstrike, Medal of Honor, Fortnite, The Witcher, Mass Effect, Fallout 4, GTA, Assassin’s Creed, Tomb Raider, Skyrim, and The Last of Us.

### Measures

**Fig 1:**
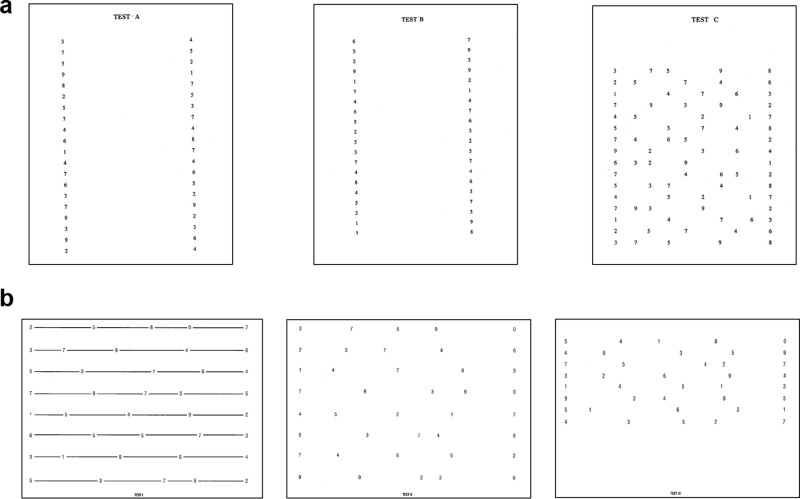
a) The DEM test includes three subtasks: tests A and B are administered in the vertical direction (up to down), and test C is administered in the horizontal direction (left-to right), b) The King‒Devick test includes three subtasks that are administered from left to right as quickly and accurately as possible. The time and errors are measured for each subtask in both tests.

All ninth and tenth graders (aged 15–16 years) from Institute “El Cairat” (Esparraguera, Spain) were screened for eligibility. After the inclusion criteria from the videogame questionnaire and visual examination, two trained optometrists performed the testing in a quiet room at the school during regular school hours. Oculomotor performance was evaluated using DEM (Bernell, Indiana, USA, Fig 1a) and the King–Devick tests (King-Devick technologies, Illinois, USA, Fig 1b). Both tests evaluate the response time during reading of digits arranged in horizontal or vertical arrays. In the DEM test, participants were asked to read the digits as fast as possible, at a constant distance of 0.4 m, seated at a table, without touching the test and keeping their heads still.

Time was assessed using a smartphone, and errors were manually marked in a scoresheet. The DEM test uses an adjusted time that is obtained by computing the errors and time using the equation: adjusted horizontal time = Test C time × [80/ (80 – o + a)], where ‘Test C time’ is the raw horizontal time, ‘o’ represents omissions, ‘a’ represents additions, and 80 is the total number of items displayed on the page (see Fig 1a). Prior to reading from DEM test pages B and C, participants were asked to read digits from the pre-test page which contains a horizontal row of numbers. Vertical time is not adjusted because errors are infrequent, [40] and the total vertical time is calculated by adding the scores for DEM parts A and B (Fig 1a). In addition, the DEM ratio is computed by dividing the adjusted horizontal time by the adjusted vertical time, providing information about the visuo-verbal responses. The test normative data applies up to age 13 years old.

The King–Devick test was conducted similarly to the DEM test because both share the same timing dynamics for reading the entire sequence of digits aloud. However, the King–Devick test was conducted on a tablet device and did not include a vertical task. Therefore, the ratio of horizontal to vertical saccade times was not calculated. The King-Devick score was calculated using the raw time in seconds, with 1 second added for each error. The order of administration of the test type (DEM vs. King-Devick) was randomized for each participant. A simple randomization process using Google’s randomizer, set to a minimum of 1 and a maximum of 2, was used to select the test order. The test administrators were masked to participant’s test order assignments.

### Statistical analysis

The main outcome measures for this study were the performance on the DEM (horizontal, vertical, ratio) and the King–Devick tests (measured in seconds). Based on the results of the video game questionnaires, participants were divided into AVG and NVG video game groups. For the DEM test, adjusted times for horizontal part were calculated following the manual. For the King–Devick test, the total time sum for the three test pages was taken into account for each analysis. Data distribution was evaluated using the Kolmogorov–Smirnov normality test with the Lilliefors correction. Parametric (unpaired t-student test) or non-parametric (Mann-Whitney “U” test) tests were used to compare between groups. In addition, a parametric ANOVA or non-parametric equivalent (Kruskal-Wallis) was performed to compare the responses between the AVG and NVG groups. Correlations between DEM and King–Devick test were determined using parametric (Pearson) or nonparametric (Spearman rho) tests. IBM SPSS 27.0 for Windows was used for statistical analysis. A significance level (p-value) of 0.05 was considered significant. Sample size was calculated using G*Power software (version 3.1.9.7; Heinrich-Heine-Universität Düsseldorf) [41]. The minimum sample size required when comparing two populations using independent means test, for a power (1-ß) of 88%, error probability of 5% (error 0.05), and to observe a difference of at least 8 seconds with 9 of standard deviation, was a total 40 participants, 20 in each group.

## Results

**Fig 2:**
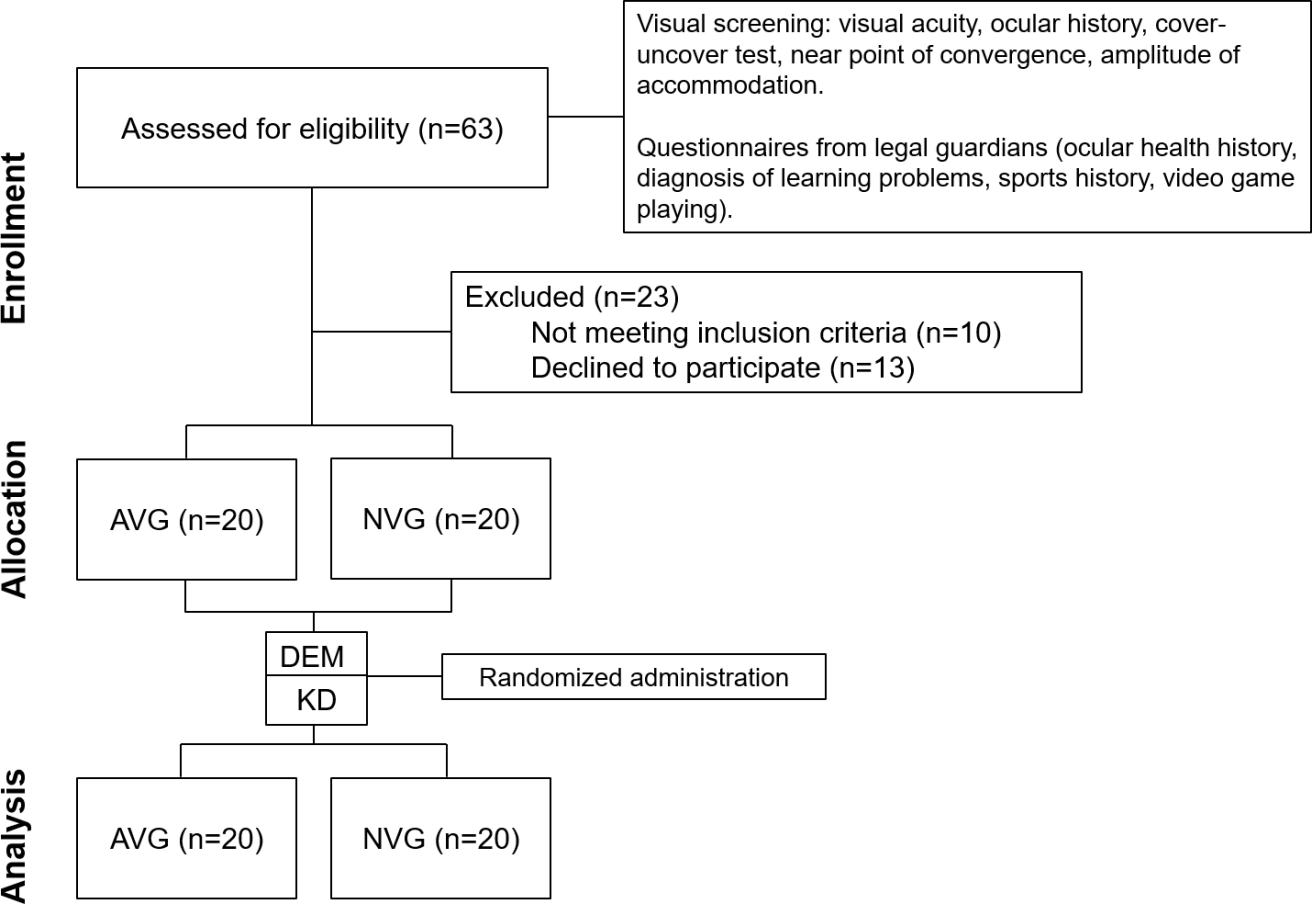
Flowchart of participants.

A total of 63 participants were screened over two months. The final study sample consisted of 40 participants, including 12 girls (30%) and 28 boys (70%) whose mean age and standard deviation (±) was 15.7 ± 0.1 years. The flowchart of participants’ enrollment, allocation, and analysis is shown in Fig 2. Mean binocular visual acuity for the entire sample was 0.99 ± 0.02 decimal, and 0.99 ± 0.02 for the right and left eyes, respectively. Mean near point of convergence was 3.67 ± 2.14 cm, and amplitude of accommodation was 15.12 ± 5.17 D, and 14.97 ± 5.35 D for the right and left eyes, respectively. The mean near phoria was 0.25 ± 0.49 prism diopters. The demographic characteristics and ocular evaluation of the AVG and NVG subgroups is shown in Table 1.

**Table 1:** Demographic characteristics of the action video game (AVG) and non-action video game (NVG) sub-groups included in the study. NPC= Near Point of Convergence (cm), AA = Amplitude of accommodation (sphere diopters, “D”), near phoria in prism diopters, “Δ”). RE = Right Eye, LE = Left Eye.

	AVG	NVG
**Participants (N)**	20	20
**Girls (N,%)**	2 (10%)	13 (65%)
**Boys (N,%)**	18 (90%)	7 (35%)
**Mean Age (years)**	15.7 ± 0.1	15.6 ± 0.1
**NPC (cm)**	3.20 ± 1.82	4.15 ± 2.36
**AA (D) RE**	16.86 ± 5.64	13.38 ± 4.07
**AA (D) LE**	16.57 ± 5.93	13.37 ± 4.27
**Near phoria (Δ)**	0.15 ± 0.36	0.35 ± 0.58

### Data is presented as means and standard deviation

Table 2 shows the DEM and the King–Devick test results of the sub-groups. Vertical DEM time was not significantly different between the sub-groups, one-way ANOVA, F(1,39)=3.65, p = 0.063, η_p_^2^ = 0.08, mean difference 2.55 ± 1.33 s. There was a significant difference in the adjusted horizontal DEM time between the sub-groups, F(1,39)=15.01, p < 0.001, η_p_^2^ = 0.28, mean difference 7.68 ± 1.98 s, with the AVG group demonstrating faster times than the NVG group. The DEM ratio was also significantly different between the sub-groups, F(1,39)=8.34, p < 0.006, η_p_^2^ = 0.18, mean difference −0.17 ± 0.05. A two-way ANOVA conducted using vertical and horizontal DEM performance showed a statistically significant difference for the horizontal time, F(1,40)=15.01, p < 0.001, but not the vertical time, F(1,40)=3.65, p = 0.063, η_p_^2^ = 0.08.

**Table 2:** Mean and standard deviation (±) in oculomotor performance in the DEM and King–Devick test (s) of the action video game (AVG) and non-action video game (NVG) participants.

Oculomotor performance			
	AVG	NVG	p-value
**Vertical DEM**	25.74 ± 3.19	28.29 ± 5.03	0.063
**Horizontal DEM**	26.15 ± 4.85	33.83 ± 7.42	<0.001
**Ratio DEM**	1.02 ± 0.19	1.19 ± 0.17	0.006
**King–Devick Test**	13.49 ± 1.93	15.68 ± 4.00	0.034

**Fig 3:**
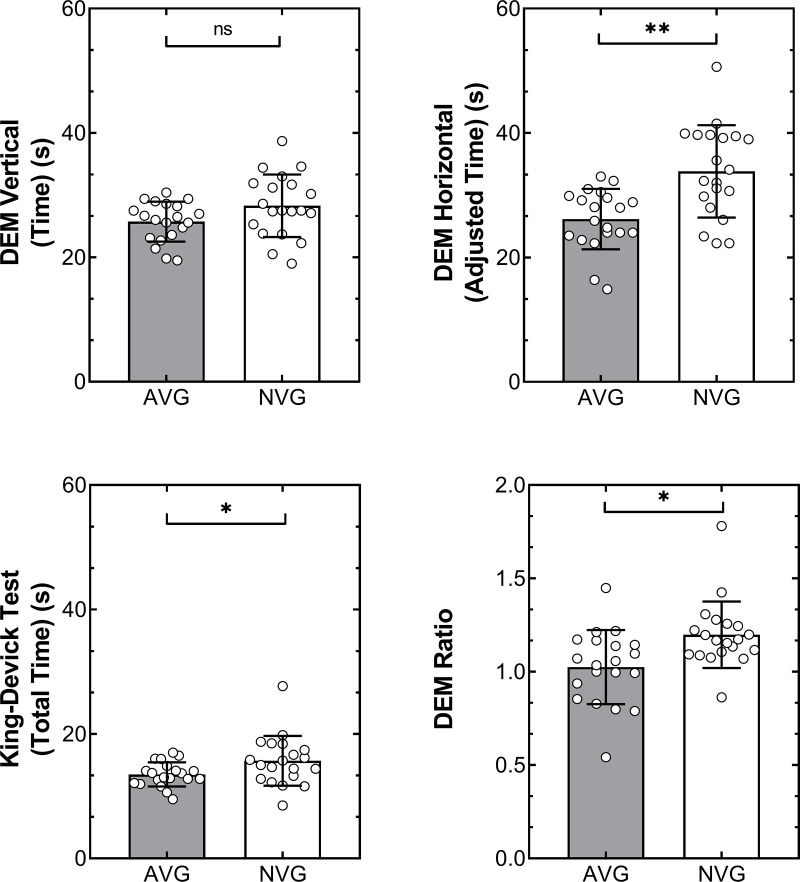
Oculomotor performance in DEM (vertical time, adjusted horizontal time, and ratio) and King–Devick tests for AVG (filled columns) and NVG (unfilled columns) sub-groups. ns = not significant (p > 0.05), * p ≤ 0.05, ** p ≤ 0.01.

The AVG group’s total performance was significantly faster on the King–Devick test compared with the NVG group, F(1,39)=4.81, p = 0.034, η_p_^2^ = 0.11, mean difference 2.18 ± 0.99 s. Fig 3 shows the results of the DEM and the King–Devick tests for both sub-groups.

**Fig 4:**
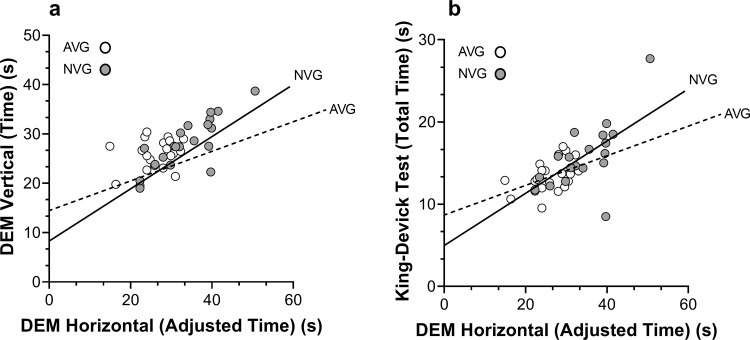
a) Correlation plot between vertical and horizontal DEM performance in the NVG and AVG groups, b) Correlation plot between total King–Devick performance and horizontal DEM performance in the NVG and AVG groups.

There was a significant correlation between vertical and horizontal DEM performance for the entire cohort, r(40)=0.68, p < 0.01, and the NVG sub-group, r(20)=0.80, p < 0.01. However the correlation between vertical and horizontal DEM performance was not significant for the AVG sub-group, r(20)=0.27, p = 0.255 (Fig 4a). A significant correlation was found between total King–Devick performance and horizontal DEM performance for the entire cohort, r(40)=0.67, p < 0.01, and between the NVG and AVG sub-groups, r(20)=0.68, p < 0.01, and r(20)=0.45, p = 0.044, respectively (Fig 4b).

## Discussion

This study compared the performance of action video game teen-aged players with non-video game teenagers on the clinical DEM and King–Devick tests. The AVG group showed significantly better results in the horizontal DEM ratio and the overall King–Devick completion time, both of which measure the performance in oculomotor and visuoverbal processes.

The results of the DEM test in the sub-groups of the present study are similar to the USA norms, and better than those reported by Fernandez-Velazquez (1995) in the Spanish population in participants aged 6–11 years [42]. These differences may be due to the fact that the present study included older participants. One limitation for this study is the use of DEM in a population of teenagers older than 13 years of age. The application of the DEM in this study is justified by the research design, which focuses on comparing performance across groups based on video game playing history rather than assessing individual performance against normative data. Further, previous studies utilized the DEM test in adult populations, [40] including individuals with traumatic brain injury, [43] demonstrating its applicability beyond the normative age range. Future studies could consider the adult DEM (A-DEM) which was developed to evaluate the oculomotor performance for ages 14–68 years old, [44] which has been reported to be correlated with DEM but not interchangeable with the DEM [45].

Previous studies examined the impact of AVG on eye movements used eye–tracking technology and adult populations [16]. Eye movement metrics in saccades, such as speed, [20] latency, [46] and fixation duration, [47] have been extensively investigated. Despite their widespread use in research, eye-tracking methodologies are also subject to inconsistencies due to factors such as hardware limitations, calibration variability, and individual differences in oculomotor behavior. These constraints reduce the reliability of eye-tracking as a tool for assessing nuanced differences in visual attention and processing due to AVG playing [48]. Hence, the novelty of this study lies in investigating the association of Action Video Game (AVG) play frequency with performance on clinically common and widely available tests, such as the Developmental Eye Movement (DEM) test and the King–Devick test, in a teenage population. These tests are more readily available than eye-trackers. Further, Ayton et al. (2009) suggested that the DEM test may not directly correlate with eye movement parameters, although they are related to other visual skills of reading performance, such as visual spatial attention, which could be useful for a diagnostic role in clinical practice [32]. Our results are in agreement with studies reporting that regularly playing AVG improves visual spatial attention, [14,49] which is associated with better reading performance, [14] and horizontal DEM test scores [50]. Moreover, the DEM test has been shown to be a useful indicator of visual-verbal naming skills, or visual processing speed [50]. Hence, the results of this study indicate that better performance of the DEM test is associated with teenagers who regularly play AVG.

The significant correlations between total King–Devick performance and horizontal DEM performance for the entire cohort are in agreement with previous reports [40]. The mean difference in King–Devick and horizontal DEM performance was approximately 19 sec and 13 sec in the NVG and AVG sub-groups, respectively. This finding demonstrates superior capability in the crowded horizontal DEM task for the AVG players. This may be attributed to improved visual search performance in crowding tasks for AVG players [51,52]. Furthermore, AVG players also demonstrated more consistent performance compared to the NVG sub-group as indicated by lower standard deviations in both the horizontal DEM and King-Devick test times.

The differences in response times for the AVG and NVG sub-groups for the horizontal DEM test (mean difference≈ 7 s) were 3.5 times higher than their differences for the King–Devick test (≈ 2 s). Although these differences in response times for the sub-groups were significantly different for both tests, the mean differences for the King–Devick test are not clinically significant as they are lower than the standard deviation. Thus, based on the results of the present study, the improved performance of regularly playing AVG only transfers to horizontal DEM performance.

One limitation for this study includes the sample size. Due to the strict inclusion criteria for visual examination and AVG history of videogame playing, only 20 were included in the AVG, and 20 in the NVG. Post-hoc power calculation using G*Power software (version 3.1.9.7; Heinrich-Heine-Universität Düsseldorf), based on the computed Cohen’s d for adjusted horizontal time DEM (d = 1.22), error probability of 5%, and 20 participants in each group, yielded a power of 96.52% for the adjusted horizontal time in DEM test. For the King-Devick test, the Cohen’s d was 0.69, and the power achieved was 57.50%. The lower power observed for the King-Devick test, despite using the same sample size and error probability as the DEM test, can be attributed to the fact that this test is easier in terms of visual processing and less sensitive in detecting associations with a history of AVG playing, compared with the DEM test.

Both the DEM and King-Devick tests have demonstrated low test-retest reliability and a strong learning effect [33,53]. For the DEM test, particularly in children from second to fifth grade, a difference of at least 12 s between the test and retest is required to exceed the repeatability threshold, as reported by Facchin and Maffioletti (2018) [33]. In our study, we found a difference of about 7 s between the AVG and NVG groups. While this suggests a trend, the measure may still be subject to variability and learning effects, which could influence result consistency across repeated administrations and replication studies.

In addition, although various commercial video games are grouped into the same genre, some video games can have distinct features related to motor, perceptual, and attentional domains [8,51]. However, while certain genres exhibit significant differences from one another, others share overlapping characteristics. This justified the inclusion of various action video games within the same genre, as was used in previous studies [5–7,54,55]. For example, action video games (AVGs) place greater demands on rapid attention shifts and motor planning than other genres. The present results suggest that future research should explore how various characteristics of action video games (AVGs), such as reaction time and motor skills, influence performance on the DEM and King–Devick tests. Additionally, it would be valuable to investigate the potential relationship between playing other video game genres (e.g., Sports, Action, Multiplayer Online Battle Arena, Role-Playing Games, Simulators, Music, Adventure) and performance on these tests.

The present results imply that future studies should examine the impact of playing AVG in the performance of DEM and King–Devick tests. In addition, there are other videogame genres who may potentially enhance visual attention abilities [56]. The possible contributions and transfer of enhanced performance to the DEM and King–Devick tests due to other videogame genres requires further research. Based on the eligibility criteria for this study and across the 63 students screened, of the 40 that were eligible for inclusion, there were only two girls in the AVG group, compared with 13 in the NVG group. This sex bias is a potential study limitation which is common in this area of research as most studies of populations who are active in AVG include more males [4].

## Conclusions

This study demonstrates that teenagers who regularly play action video games exhibit superior performance in the DEM and King–Devick tests, which assess oculomotor skills and visual processing speed. Clinicians administering these tests should consider the potential influence of habitual action video game use when interpreting results, as it may reflect an elevated performance of these abilities rather than solely indicating developmental or clinical concerns.

## Supporting information

S1 File Inclusivity in global research. (DOCX)
